# Technology transfer, intellectual property, and the fight for the soul of WHO

**DOI:** 10.1371/journal.pgph.0003940

**Published:** 2024-12-05

**Authors:** Melissa Barber

**Affiliations:** Yale Law School, Yale School of Medicine, Yale Collaboration for Regulatory Rigor, Integrity, and Transparency, New Haven, Connecticut, United States of America; London School of Economics (LSE), UNITED KINGDOM OF GREAT BRITAIN AND NORTHERN IRELAND

## Abstract

Debates over the scope, terms, and governance of technology transfer–the sharing of essential technical information, know-how, and materials needed to manufacture a health product–are prominent and controversial in international health diplomacy. These debates have become focal points in recent contentious negotiations to amend the International Health Regulations (IHR) and draft a global Pandemic Agreement. While some countries advocate for automatic or compulsory mechanisms to facilitate access to health technologies, especially in times of crisis, others oppose legal frameworks that mandate non-voluntary participation by the pharmaceutical industry. Also at stake are questions of institutional mandate: the United States has amplified calls by industry that pandemic technology transfer policy should be the domain of the World Trade Organization (WTO) instead of the World Health Organization (WHO). This essay offers a counternarrative to claims that WHO is overstepping its historic role in global governance. Far from being a contemporary development, technology transfer was at the heart of WHO’s work at its founding. WHO’s early failure to secure antibiotic technology transfer in the face of US opposition led to its first major crisis, prompting the withdrawal of several member states. In response, WHO embarked in the 1950s on a visionary programme to establish a global network of non-profit, state-run drug manufacturers and scientists committed to the free exchange of knowledge. This ambitious initiative has been largely forgotten, excluded even from WHO’s self-published accounts of historical technology transfer work. In the context of ongoing pandemic governance negotiations and the nascent mRNA hub program, remembering the lost vision of global solidarity embodied in WHO’s midcentury technology transfer program offers a glimpse into an alternate path we might still chart, one where access to medicines is not bound by the logic of enforcing scarcity to maximize profit, and the right to health is a global responsibility.

## Introduction

At the May 2024 World Health Assembly (WHA), the 194 Member States of the World Health Organization (WHO) passed the first amendments to the International Health Regulations (IHR) since 2005 [1]. The amendments provide definitions for events that would trigger pandemic responses under the framework, add equity and solidarity as guiding principles, and commit WHO to establishing and coordinating "mechanisms […] that facilitate timely and equitable access to relevant health products", including those that enable domestic or regional manufacturing for key health products [2]. Such mechanisms are considered crucial to mitigate the risks of globalized supply chains, for example, where disruptions at a one factory can trigger global shortages [3].

The adoption of the IHR amendments was not without controversy: Slovakia disassociated from the resolution and Argentina, Iran, and Russia stated that national sovereignty would come before international agreements [4]. Conspicuously absent in the final text of the IHR amendments were provisions addressing technology transfer and intellectual property; agreement could not be reached even on the language of the relevant footnote [2, 5]. Sharply divergent views on whether technology transfer–the sharing of essential technical information, know-how, and materials needed to manufacture a health product–should be initiated without the voluntary consent and participation of the technology holder have similarly contributed to the impasse in negotiations on the global Pandemic Agreement, which were supposed to wrap up mid-2024 but instead have been extended by at least a year [2, 6]. It remains to be seen if consensus will be reached in 2025, or negotiations will have to be further extended.

Underlying heated technical debates is a simple question: who should manufacture health products and who should have priority to receive scarce supply in a public health emergency?

Today, WHO is largely a technical institution, providing normative guidance and at most, serving as a neutral convener for other institutions to promote technology transfer and manufacturing [7–9]. Although technology transfer has been identified as critical to improving the world’s ability to respond to pandemics, WHO has in recent decades played a limited role. But in the first years after the creation of the WHO in 1948, WHO and UNICEF led a bold campaign to expand manufacturing capabilities for new health technologies globally and to establish publicly-owned factories. In contrast to the small-scale technology programs for palivizumab, influenza vaccines, and vaccine adjuvants of the last two decades, the 1950s program aimed at nothing less than a global network of non-profit drug manufacturers committed to open science [8]. This ambitious initiative has been largely forgotten, excluded even from a history of WHO technology transfer hubs published by the *Bulletin of the World Health Organization* [8]. In the wake of the passage of the underwhelming IHR Amendments and ongoing negotiations for the Pandemic Agreement [10], remembering the lost vision of global solidarity embodied in WHO’s 1950s technology transfer program offers a glimpse into an alternate path we might still chart, one where access to medicines is not bound by the logic of enforcing scarcity to maximize profit, and the right to health is a global responsibility.

### The antibiotic revolution and global health diplomacy

WHO was created as a specialized agency of the United Nations in 1948, just four years after the start of industrial production of penicillin, one of the most significant medical advances of the century. Although Fleming discovered the antibiotic properties of *Penicillium* in 1928, chemists were unable until the early 1940s to produce enough antibiotic even for a clinical trial [11, 12]. Penicillin supply remained so scarce that penicillin was extracted from treated patients’ urine and reused until the mid-1940s, when a secret United States government research program–medicine’s version of the Manhattan Project–advanced fermentation technology enough to facilitate industrial-scale production [11–13]. During the war, the United States had taken a pragmatic approach to intellectual property, effectively issuing compulsory licenses by forcing private companies to license penicillin-related patents to the government [14]. After the war, an international agreement was struck dividing patent rights among participating firms, while still granting non-exclusive royalty-free licenses to the United States and United Kingdom governments [15].

After the secrecy around the project was lifted at the close of World War II, scientists involved in the penicillin development program were eager to expand access globally. Their enthusiasm was tempered by uncertainty as to if and how pharmaceutical companies would enforce patents relating to its production processes globally [15]. In January 1948, three months before WHO’s official establishment, the first WHO Expert Committee on Venereal Diseases recommended that “measures should be taken to encourage production of penicillin [and] equitable distribution to all countries” [16]. In January 1949, WHO held a summit with Byelorussia, Czechoslovakia, Poland, Ukraine, and Yugoslavia to develop plans for building or modernizing factories [17, 18]. Operations were halted when the United States refused to grant WHO export licenses on the Podbielniak extractors essential for manufacturing antibiotics [17].

It was in this tense context of WHO being blocked by its own Member State from purchasing essential equipment that its governing body, the World Health Assembly (WHA), held its second meeting in June 1949. Poland submitted an agenda item condemning WHO Member States that were keeping antibiotic manufacturing processes secret, arguing that “such behaviour is not in accordance with the Constitution of the World Health Organization which was signed by these countries” [19]. Poland found many allies in grievance, with Czechoslovakia and El Salvador joining them in expressing concern about secrecy in medical research limiting WHO’s ability to deliver on its promises to support countries in establishing antibiotic production. Resolution WHA2.44 stopped short of explicitly condemning Member States for secrecy, but reiterated the WHO’s position that “withholding of scientific or technical information on essential therapeutic and prophylactic drugs” was not compatible with WHO’s ideals and was “against the interests of humanity” [20]. Delegates from the United States were “startled and depressed at the attack”, and in a move not dissimilar from today, sought to insulate themselves from policy critique by emphasizing their role as WHO’s leading donor [21].

### The first crisis

By August 1950, eight countries had announced they were withdrawing from WHO. Chief among their complaints was WHO’s failure to deliver on technology transfer. For example, Romania criticized WHO for failing to “[contribute] in a satisfactory manner to the dissemination of scientific progress in medicine”[22]. Poland explicitly referred to WHO’s impotence in purchasing Podbielniak extractors, condemning the United States and WHO for failing to challenge the United States, “thus prejudicing the WHO basic principle that each country may participate in the results obtained anywhere in the field of health production” [23].

Technology transfer was no longer just a public health priority, but existentially vital to WHO’s legitimacy as a global organization for health. WHO established an Expert Committee on Antibiotics in 1950 [24]. It is clear from the earliest days that members of the committee understood both the public health importance of disseminating penicillin technology and the political opposition they would face. In a draft copy of their first report, the only sentence to be crossed out recalls that the proposed production program was mandated by the first World Health Assembly, which called for “all possible measures [to] be taken by WHO to encourage antibiotics production and to ensure an equitable distribution to all countries” [24]. This sensitivity around recalling a controversial mandate will be familiar to anyone who has participated in text-based negotiations: although the proposed program of work remained untouched, the report’s drafters may have sought to deflect unwanted attention by softening the document’s sharper, more polemical edges.

Opposition from the United States and pharmaceutical companies holding patent rights related to antibiotic technology was swift. The chair of the Antibiotic Committee, Ernst Chain–by then a Nobel Prize winner for his contributions to the development of penicillin–was refused a visa for an official WHO mission to the United States [25]. In a confidential, personal letter, Director-General Brock Chisholm apologized to Chain that high-level efforts to persuade the United States to grant his visa had failed, describing the current atmosphere in the United States as a “somewhat hysterical tension […] towards qualifying biological information as security material” [26]. Although the State Department gave no reason, Chain believed that he was refused because of an earlier visit (also on WHO official business) to Czechoslovakia, or that “the State Department simply did not wish to encourage international collaboration in the field of antibiotics”, a hypothesis for which he claimed in a letter to the Weizmann Institute of Sciences to have evidence [25]. Merck, a pharmaceutical company that was a major beneficiary of penicillin patents, did not have representation on the Antibiotic Committee [15, 27]. Nevertheless, Merck made sure the Committee knew that close tabs were being kept on its work, writing to its chair to request a list of all known penicillin and streptomycin manufacturers, their production volumes, and an account of all facilities receiving UN assistance [28].

### Penicillin for the people

WHO’s new antibiotics department was tasked with launching an international group of collaborating researchers, training scientific personnel, and supporting governments in establishing domestic antibiotic production [29]. Countries received support to design factories and equipment, set up production lines, and procure equipment [29]. Sahib Singh Sokhey was recruited in 1950 to oversee the technology transfer project as WHO’s Assistant Director-General of Technical Services [30]. Sokhey was uniquely well-suited in both outlook and experience to lead the program [30]. He understood that WHO’s vision of “health for all” was not universally shared, and had experienced first-hand the lengths pharmaceutical corporations would go to protect intellectual property, such as proprietary manufacturing techniques. As the director of the Haffkine Institute, a research organization based in Bombay, Sokhey conducted clinical trials demonstrating that sulphathiazole was effective in reducing bubonic plague mortality from 76% to 7% in patients treated in the first 24 hours after onset [31–36]. (Far from being a terror from the distant past, bubonic plague remained endemic in India well into the twentieth century, accounting for over ten million deaths 1897–1930) [37]. The patent holder, May and Baker, had not contributed to research demonstrating novel uses of their drug, nor did they even market the drug in India [33]. Nevertheless, after the Haffkine established a small manufacturing operation to stock public health centers across India, May and Baker exerted their patent rights, fighting the Haffkine’s attempt to secure the government-use license needed to continue the manufacturing program [33]. The bleak absurdity of a pharmaceutical company prioritizing a patent-protected global monopoly over immediate need to treat the bubonic plague in an impoverished country seems to have radicalized Sokhey, who for the rest of his life advocated for intellectual property reform [34, 38, 39].

WHO’s antibiotic technology transfer and manufacturing program was governed by three principles. First, production would proceed on a non-profit basis [39]. Second, every stage would seek to use and build local capacity, with the ultimate aim of supporting countries in “eventually [becoming] self-sufficient […] without dependence on foreign help” [29, 40]. WHO set up agreements with research institutes, most notably the Istituto Superiore di Sanità in Rome, to provide training on antibiotic production technology [39, 41, 42]. Third, both scientific and industrial knowledge would be freely shared and not commercialized. A spirit of optimistic internationalism permeated the project, with each factory envisioned as “[becoming] a training ground for other plants” [29]. In sharp contrast to WHO’s contemporary position of “balancing” intellectual property and health [43], the WHO of that era proposed “national production plants operating on a non-profit basis and free from the commercial secrecy which in recent years has clouded the whole subject of antibiotic research methods” [39]. The program’s insistence that open science was feasible was accompanied by a realistic assessment of the obstacles: a flurry of correspondence among WHO staff, antibiotics committee experts, and lawyers mapping the patents covering various production technologies attest to fears that litigation by patent-holders could doom the project [44–46]

The negotiations that followed, thrillingly narrated in a 2004 history by Nasir Tyabji, were more reminiscent of John le Carré novels than routine diplomatic affairs [47]. Nehru and his deputies demanded proof that WHO’s proposed production processes would not be ensnared by patent litigation, but found WHO frustratingly reluctant to provide details about manufacturing [47]. WHO in turn was convinced that Neville N. Wadia, the chair of India’s Committee on the Penicillin Project, was reporting their every move to Glaxo and Merck, who were interested in gathering evidence for future patent disputes on what production processes WHO planned to use [47]. Through a series of backchannels, unofficial hotel meetings, and handwritten letters to avoid the risk of compromised typists, WHO was finally able to persuade Nehru of the feasibility of their proposal [47].

**Fig 1:**
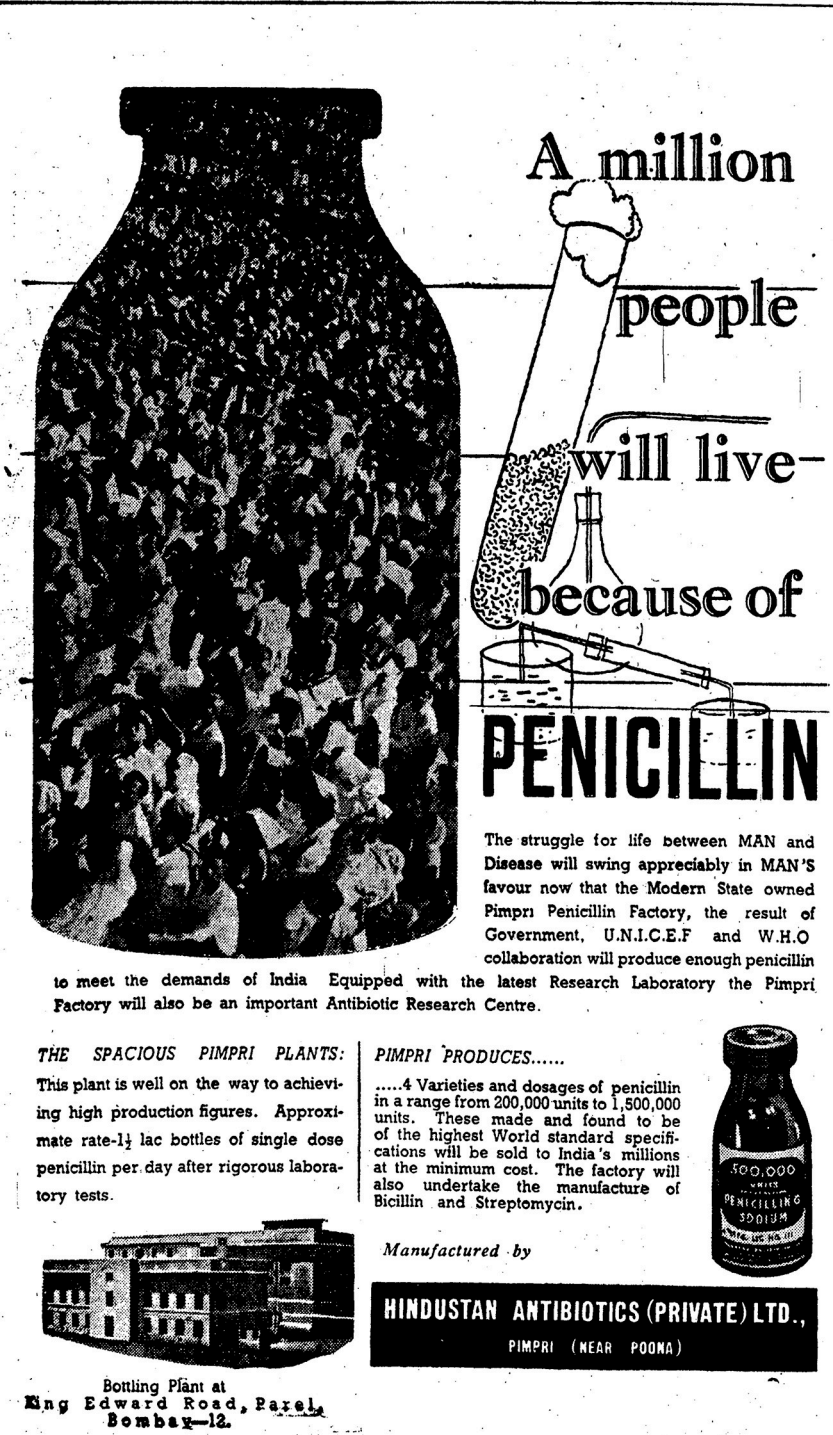
Advertisement for penicillin produced by Hindustan Antibiotics Ltd. *The Times of India*, 1956 [64].

**Fig 2:**
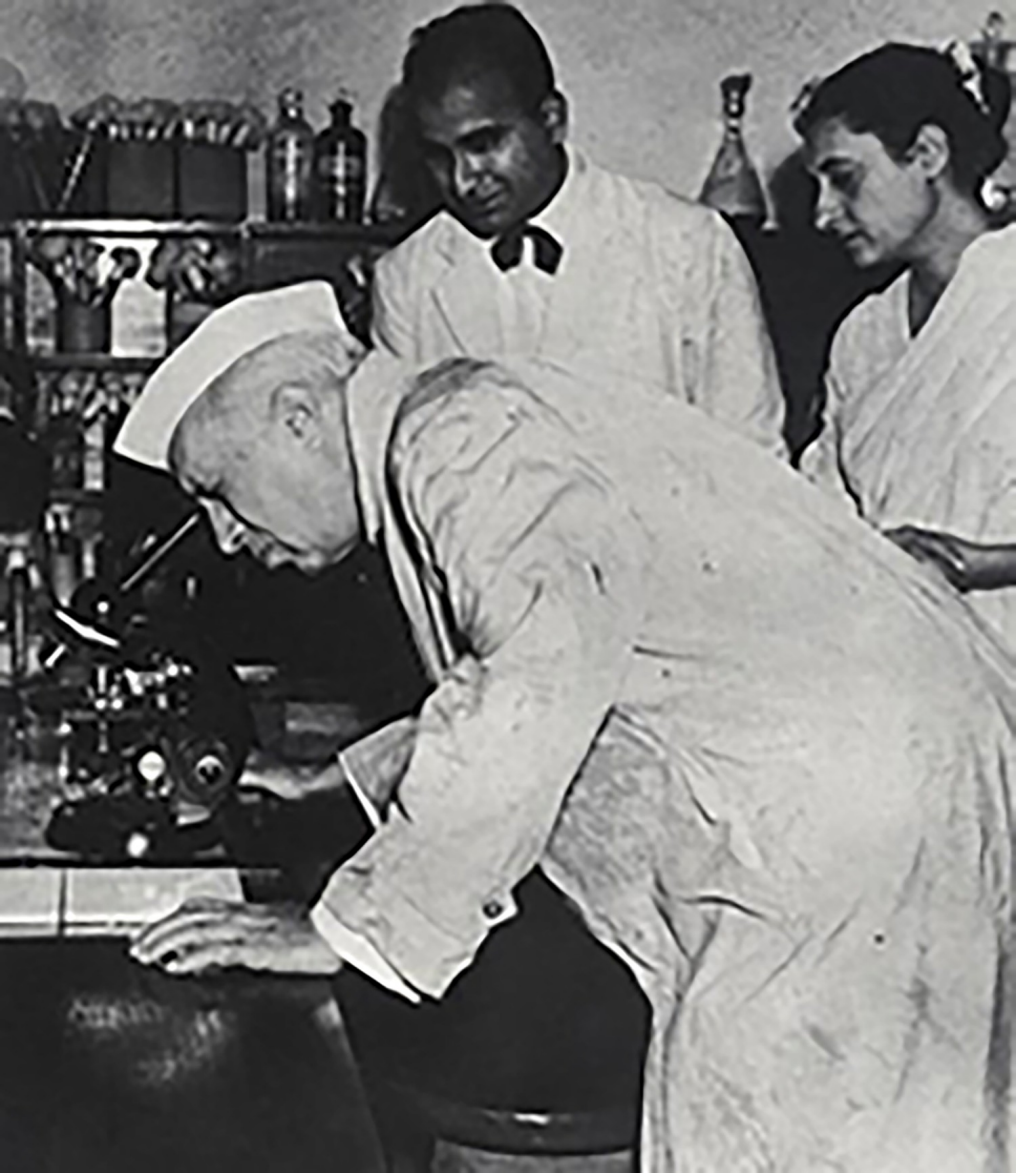
Jawaharlal Nehru at the Penicillin Factory, Poona, 1 August 1956. From: Nehru Memorial Museum & Library [65].

In 1951, India, WHO, and UNICEF finally signed an agreement to build a public penicillin factory, thereby inaugurating Hindustan Antibiotics Ltd (HAL) as the first public pharmaceutical firm in India [40]. By 1958, the HAL factory in Pimpri was producing 35 million mega-units of penicillin annually, enough to meet 70% of India’s total 50 million mega-unit consumption (Fig 1) [48]. In a speech on the first anniversary of the factory’s opening, Nehru reflected on the importance of open science, declaring that “[s]cience did not flourish on methods of secrecy […] it was for scientists to proclaim to the world their discoveries to advance the cause of science and also the benefit mankind. It was wrong to allow private firms to keep processes of preparing medicines a secret” (Fig 2) [49].

However remarkable its establishment, the utopian experiment at Pimpri was soon bruised by opposition at home and abroad. Domestically, industrialists and anti-communists chafed at state control of a key industry [47, 50–52]. Internationally, the United States cajoled and ultimately pressured India to accept agreements with the United States-based company Merck that limited Indian scientists’ access to technical know-how and required the payment of significant royalties [53,54]. While remaining in public ownership, HAL would in the coming decades lose some of its revolutionary zeal for open science and self-sufficiency and instead gradually enter into technology transfer agreements with multinational pharmaceutical companies. In some cases, partner manufacturers followed the letter but not spirit of technology transfer contracts. As one example, HAL entered into a contract with Merck which required Merck to notify HAL of any advances in streptomycin production technology [53]. Years later, it emerged Merck had not shared an improved strain. When investigated, Merck’s defense hinged on the fact the strain was purchased for use in their labs but not developed *within* their labs, and therefore Merck was not contractually obligated to notify HAL [53]. This and similar episodes galvanized opposition to HAL’s shift to private sector partnerships. MP Rena Chakravartty highlighted the persistent problems of such agreements, asking a 1959 Lok Sabha Committee why the Indian government “rush[ed] into this [streptomycin] agreement… Did our Ministry not have any understanding of the situation? Did they not know who Mercks [*sic*] was?” [55].

By 1953, WHO’s pharmaceutical manufacturing program had “[increased] to a point where the work was much more [that] of a large-scale industrial undertaking than of a health organization service” [48]. However, in the same year, WHO suffered a “drastic cut” in funds, and the program was transferred to the United Nations Technical Assistance Administration (UNTAA) [29]. The 1953 Chronicle of the WHO notes the quiet closing of “one of the most daring and successful endeavors” [29]. WHO’s approach had diverged from the preferences of its largest donor, the United States, who favored expanding intellectual property protections and opposed public sector manufacturing. Throughout the 1940s and 1950s, the United States hindered WHO’s efforts to disseminate antibiotic technology by denying visas, blocking equipment export licenses, and pressuring India to refuse WHO’s proposal and instead accept a contract with Merck [17, 26, 56]. In contrast, although the UNTAA continued to provide limited technical support to countries, it did not pursue the earlier WHO program’s radical vision of open science, international solidarity, and public manufacturing [29, 57]. Declassified policy memos reveal that the United States primarily viewed the UNTAA as a means to defuse pressure from African, Asian, and Latin American countries, who called for redistributive poverty reduction funds that would provide capital investments for public infrastructure, rather than UNTAA’s conservative provision of technical experts [57, 58].

### Lessons learned from a compromised vision for technology transfer

What lessons are there to be drawn from this daring project? On the one hand, the WHO antibiotics technology program offered an alternative vision of global health internationalism by demonstrating that technology transfer models grounded in public-sector leadership and open science rather than commercialization and voluntary measures could be successful. On the other hand, the obstacles the program faced and its untimely demise warn of the immense challenges faced by global health initiatives attempting to reshape capital to meet global health needs.

The unmet need for technology transfer has always simmered below the surface of global health governance debates. Far from being beyond WHO’s purview (as recently suggested by Ambassador Pamela Hamamoto, the United States representative to the Pandemic Agreement negotiations), tensions surrounding intellectual property and technology transfer permeated WHO’s founding [59]. Their importance is underscored by the fact that WHO nearly fell apart before it had begun, in no small part because of its inability to deliver on the technology transfer demanded by some Member States and obstructed by others.

In response to the COVID-19 pandemic, WHO has increased its focus on technology transfer, both through its mRNA technology transfer hub, and through its work on related international agreements. Regrettably, the threats to these initiatives remain much the same as those in 1950. Private manufacturers are reluctant to share technology in general, and especially through multilateral mechanisms: a single company has participated in WHO’s COVID-19 Technology Access Pool (C-TAP) [60]. The proliferation of public-private partnerships in the last 40 years has constrained countries’ freedom to participate in international technology sharing initiatives. As one example, Brazil’s state-owned company Bio-Manguinhos cannot fully participate in the mRNA hub because of pre-existing, exclusive technology transfer agreements with AstraZeneca [61]. Perhaps most importantly, looming over budgets and planning is the threat of future litigation over mRNA technology by patent holders, as well as trade pressure by their governments [62].

The myth of WHO as a consensus-based system obfuscates the fact that, while trade and public health are not strictly a zero-sum game, there are nevertheless winners and losers. The ideological hegemony that public-private partnerships have enjoyed in recent decades as the solution to all development problems has made it difficult for public health actors to make the case for public-public partnerships [63]. After all, who could oppose drawing from the broadest possible network of resources and expertise? But there are also costs: industry is more likely to agree to participate in and less likely to sabotage institutional configurations that are commercially beneficial. In this way, the widely held view that public-private partnerships are less risky becomes a self-fulfilling prophecy that forecloses some possibilities for structural change. WHO’s public antibiotic program was perhaps so short-lived not because its model was intrinsically unviable but because it was so successful, and by extension, threatening to those benefiting from the enclosure and privatization of pharmaceutical production know-how.

In the context of ongoing Pandemic Agreement negotiations, remembering the WHO’s antibiotics program holds a mirror to just how far aspirations for global health multilateralism and solidarity have shrunk. Historical amnesia about this once-prominent program–WHO leaves it out of its published history of technology transfer efforts–is a testament to how historical memory is forged by present political realities. When past, equity-driven models for drug access become unrememberable as legitimate policy precedents, the field of vision for viable future options to address pandemics and other health challenges narrows. Revisiting this history is therefore more than an act of memory: it calls into question the inevitability of the prevailing model of innovation and access–one which relies on artificial scarcity and inequity–and should inspire us to reconsider alternative models of international cooperation to realize drug access for all.
